# Quantitative trait loci identified for blood chemistry components of an advanced intercross line of chickens under heat stress

**DOI:** 10.1186/s12864-016-2601-x

**Published:** 2016-04-14

**Authors:** Angelica Van Goor, Christopher M. Ashwell, Michael E. Persia, Max F. Rothschild, Carl J. Schmidt, Susan J. Lamont

**Affiliations:** Department of Animal Science, Iowa State University, Ames, IA USA; Department of Poultry Science, North Carolina State University, Raleigh, NC USA; Department of Animal and Poultry Sciences, Virginia Polytechnic Institute and State University, Blacksburg, VA USA; Department of Animal and Food Sciences, University of Delaware, Newark, DE USA

## Abstract

**Background:**

Heat stress in poultry results in considerable economic losses and is a concern for both animal health and welfare. Physiological changes occur during periods of heat stress, including changes in blood chemistry components. A highly advanced intercross line, created from a broiler (heat susceptible) by Fayoumi (heat resistant) cross, was exposed to daily heat cycles for seven days starting at 22 days of age. Blood components measured pre-heat treatment and on the seventh day of heat treatment included pH, pCO_2_, pO_2_, base excess, HCO_3_, TCO_2_, K, Na, ionized Ca, hematocrit, hemoglobin, sO_2_, and glucose. A genome-wide association study (GWAS) for these traits and their calculated changes was conducted to identify quantitative trait loci (QTL) using a 600 K SNP panel.

**Results:**

There were significant increases in pH, base excess, HCO_3_, TCO_2_, ionized Ca, hematocrit, hemoglobin, and sO_2_, and significant decreases in pCO_2_ and glucose after 7 days of heat treatment. Heritabilities ranged from 0.01-0.21 for pre-heat measurements, 0.01-0.23 for measurements taken during heat, and 0.00-0.10 for the calculated change due to heat treatment. All blood components were highly correlated within measurement days, but not correlated between measurement days. The GWAS revealed 61 QTL for all traits, located on GGA (*Gallus gallus* chromosome) 1, 3, 6, 9, 10, 12–14, 17, 18, 21–28, and Z. A functional analysis of the genes in these QTL regions identified the Angiopoietin pathway as significant. The QTL that co-localized for three or more traits were on GGA10, 22, 26, 28, and Z and revealed candidate genes for birds’ response to heat stress.

**Conclusions:**

The results of this study contribute to our knowledge of levels and heritabilities of several blood components of chickens under thermoneutral and heat stress conditions. Most components responded to heat treatment. Mapped QTL may serve as markers for genomic selection to enhance heat tolerance in poultry. The Angiopoietin pathway is likely involved in the response to heat stress in chickens. Several candidate genes were identified, giving additional insight into potential mechanisms of physiologic response to high ambient temperatures.

**Electronic supplementary material:**

The online version of this article (doi:10.1186/s12864-016-2601-x) contains supplementary material, which is available to authorized users.

## Background

Climate change has increased the frequency of severe heat waves and the global temperature is projected to become increasingly warmer [1]. Heat stress in poultry negatively impacts animal production and welfare resulting in economic losses estimated to be between $125-165 million for the U.S. broiler poultry industry [2]. During a severe heat wave in Iowa, over 1.5 million layer hens died [3].

To reduce core body temperature during periods of heat stress, blood flow to internal organs decreases and blood flow to the combs and other surface tissues increases in chickens [4]. During periods of heat stress, blood volume and oxygen carrying capacity are altered [5] and dehydration, caused by increased respiration, can increase hematocrit [6]. Energy availability, as determined by plasma glucose level, is increased in chickens exposed to heat stress [7].

During high ambient temperatures, chickens reduce feed intake by as much as 17 %, which reduces growth [8]. However, metabolic and endocrine changes during heat stress also contribute to reduction in growth in broilers, as demonstrated by a pair-feeding study [9].

A major change in blood components is caused by heat-induced increased respiration, which results in respiratory alkalosis, a disturbance in the acid base balance characterized by an increase in blood pH accompanied by a decrease in pCO_2_. Respiratory alkalosis occurs in broilers during heat stress and is associated with reduced growth rate [10]. Metabolic alkalosis is an additional measure of disturbances in acid base balance and is defined by a decrease in the fixed acid concentrations and an increase in fixed base concentrations within the extracellular fluid [11].

Electrolyte balance is essential for acid base balance, maintenance of cellular homeostasis, synthesis of tissue protein, electrical potential of cell membranes, enzymatic reactions, and maintaining osmotic pressure [12]. Altering electrolyte amounts in feed partially ameliorates the negative impacts of heat stress in broiler chickens [13].

The goal of the current study was to identify the physiological changes and genomic regions associated with response to heat stress in chickens as characterized by the blood chemistry components, including pH, pCO_2_, pO_2_, base excess (BE), HCO_3_, TCO_2_, K, Na, ionized Ca (iCa), hematocrit (Hct), hemoglobin (Hb), sO_2_, and glucose (Glu). In a commercial egg laying population, developmental measures have been established with hopes of using measures of blood chemistry components for selection [14]. To date, few studies have identified quantitative trait loci (QTL) for blood components in chicken [15–18]. We used a 600 K SNP panel to identify QTL regions associated with levels of blood components of chickens under thermoneutral and heat stress conditions, and changes induced by heat.

## Results

### Blood component measurements and heritabilities

Phenotypic means and heritabilities are given in Table 1 for blood components measured pre-heat (day 20 of age), after 7 days of heat treatment (day 28 of age), and the calculated change due to heat treatment (day 28–20). After 7 days of heat treatment, pH, BE, HCO_3_, TCO_2_, iCa, Hct, Hb, and sO_2_ significantly increased while pCO_2_ and glucose significantly decreased. There were no significant changes in pO_2_, K, and Na due to heat treatment.

**Table 1:** Phenotypic means and heritabilities (h^2^)

Trait	Day 20		Day 28		Day 28-20	
	Mean ± SEM	h^2^ (SE)	Mean ± SEM	h^2^ (SE)	Mean ± SEM	h^2^ (SE)
pH	7.50 ± 0.0^a^	.17 (0.08)	7.53 ± 0.003^b^	.10 (0.08)	0.03 ± 0.004	.05 (0.03)
pCO_2_, mmHg	31.9 ± 0.1^a^	.21 (0.06)	31.1 ± 0.2^b^	.05 (0.04)	−0.8 ± 0.2	.07 (0.05)
pO_2_, mmHg	43.3 ± 0.3^a^	.06 (0.04)	43.9 ± 0.2^a^	.05 (0.05)	0.5 ± 0.3	.00 (0.03)
BE, mM	1.8 ± 0.1^a^	.10 (0.05)	3.3 ± 0.2^b^	.02 (0.02)	1.5 ± 0.2	.00 (0.02)
HCO_3_, mM	25.0 ± 0.1^a^	.05 (0.04)	26.0 ± 0.1^b^	.23 (0.12)	1.0 ± 0.2	.03 (0.02)
TCO_2_, mM	25.9 ± 0.1^a^	.02 (0.03)	26.9 ± 0.1^b^	.13 (0.09)	1.0 ± 0.2	.01 (0.01)
K, mM	4.8 ± 0.0^a^	.20 (0.01)	4.9 ± 0.0^a^	.02 (0.01)	0.1 ± 0.0	.10 (0.06)
Na, mM	137.0 ± 0.2^a^	.08 (0.6)	137.2 ± 0.3^a^	.01 (0.01)	0.3 ± 0.3	.01 (0.01)
iCa, mM	1.25 ± 0.0^a^	.04 (0.01)	1.28 ± 0.01^b^	.02 (0.01)	0.02 ± 0.01	.01 (0.01)
Hct, % PCV	22.5 ± 0.2^a^	.01 (0.03)	23.2 ± 0.1^b^	.21 (0.08)	0.7 ± 0.2	.02 (0.01)
Hb, g/dL	7.7 ± 0.1^a^	.07 (0.05)	7.9 ± 0.0^b^	.11 (0.04)	0.2 ± 0.1	.02 (0.01)
sO_2_, %	83.2 ± 0.2^a^	.03 (0.05)	84.7 ± 0.2^b^	.02 (0.02)	1.5 ± 0.3	.01 (0.01)
Glu, mg/dl	252 ± 0.8^a^	.15 (0.08)	243 ± 1^b^	.19 (0.09)	−8 ± 1	.02 (0.02)

Blood chemistry components were measured pre-heat (day 20), on the seventh day of heat treatment (day 28), and the calculated change due to heat (day 28–20). Different superscript letters within row represent significant differences (*P* ≤ 0.05)

Heritabilities ranged from 0.01-0.21 for pre-heat measurements, 0.01-0.23 for measurements taken during heat, and 0.00-0.10 for the calculated change due to heat treatment.

### Trait correlations

**Fig. 1:**
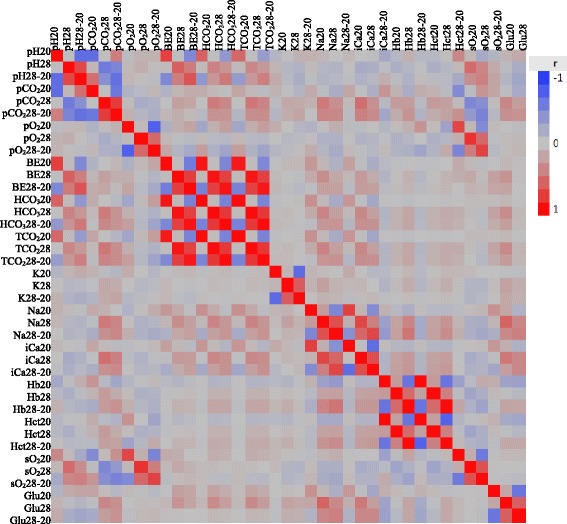
Heat map of phenotypic correlations between blood chemistry components. Heat map showing phenotypic correlations between blood chemistry components measured on day 20 (pre-heat), day 28 (during heat), and day 28–20 which is the difference due to heat treatment. Traits are clustered together based on function. The colors represent the correlation coefficient (r^2^) with *red* indicating a positive correlation and *blue* indicating a negative correlation

Correlations between blood components at each measurement phase are given in Fig. 1 as a heat map. Almost all blood components were positively correlated with all other variables measured on the same day. Very few significant correlations, however, occurred between variables measured on different days.

### Genotyping

Of the 480 genotyped birds, 458 Advanced Intercross Line (AIL) and all 12 parental line birds passed the whole animal DishQC criterion. Of the 580,961 SNPs on the array, filtering based on SNP call rate ≥ 95 % removed a small proportion (59,789 SNPs), whereas filtering based on MAF removed a much larger proportion (311,055 SNPs), yielding 210,117 SNPs for subsequent analyses.

### GWAS

**Fig. 2:**
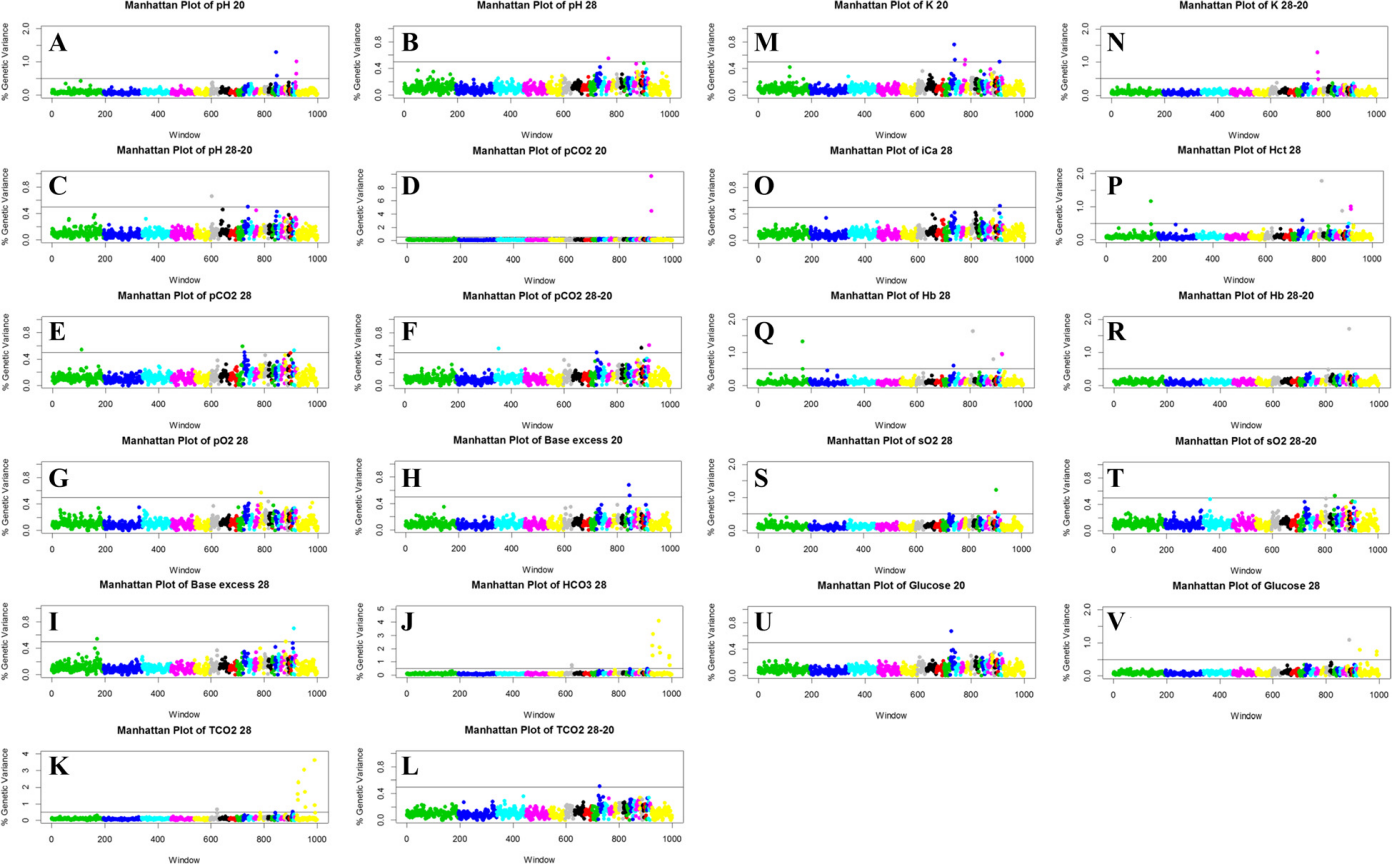
Genome-wide plot of percentage of genetic variance for traits measured during heat stress. Traits were measured before heat treatment (day 20) and during heat treatment (day 28), and the differentials were also calculated (day 28-20). The traits that reached significance in the GWAS (≥0.05 % of the genetic variation) are displayed. Results show the percentage of genetic variance that is explained by each non-overlapping 1-Mb window, labeled by the index number of the windows, and are colored and ordered by chromosome (1 to 28, and Z). Plots display: pH on days 20 and 28, and the differential 28–20 (**a**, **b**, and **c**); partial CO_2_ (pCO_2_) on days 20, 28, and the differential 28–20 (**d**, **e**, and **f**); partial O_2_ (pO_2_) on day 28, (**g**); base excess on day 20 and day 28, (**h** and **i**); bicarbonate (HCO_3_) on day 28 (**j**); total CO_2_ (TCO_2_) on day 28 and the differential 28–20 (**k** and **l**); potassium (**K**) on days 20 and the differential 28–20 (**m** and **n**); ionized calcium (iCa) on day 28 (**o**); hematocrit (Hct) on day 28 (**p**); hemoglobin (Hb) on day 28 and the differential 28–20 (**q** and **r**); saturated oxygen (SO_2_) on day 28 and the differential (**s** and **t**); glucose on days 20 and 28 (**u** and **v**)

The results from the GWAS for each trait are depicted in Fig. 2. A wide range of genetic variation (0.5-9.8 %) was explained by each significant window and detailed information is found in Table 2. Adjacent windows that were significant for a single trait are discussed below as a single QTL region.

**Table 2:** Windows explaining a significant percentage (≥0.5) of genetic variance

Windows explaining ≥ 0.5 % of genetic variance						SNP with highest model frequency within window			
Trait^a^	Chr	Pos (Mb)	% of genetic variance explained	Nb of SNPs	Freq of iterations with (*P* > 0)^b^	SNP name^c^	SNP pos (bp)^d^	Model freq^e^	Allele freq^f^
pH20	18	3	1.29	401	0.94	AX-75894740	3342614	0.0111	0.652
pH20	28	4	1.01	328	0.85	AX-76384843	4097788	0.0090	0.294
pH20	28	3	0.64	437	0.92	AX-76383580	3856132	0.0092	0.294
pH20	18	6	0.58	342	0.86	AX-75894671	6670745	0.0075	0.340
pH28	12	7	0.55	302	0.81	AX-75723368	7630857	0.0070	0.288
pH28-20	6	4	0.66	350	0.86	AX-76958371	4259110	0.0074	0.724
pH28-20	10	16	0.50	372	0.89	AX-75591175	16460945	0.0066	0.298
pCO_2_20	28	3	9.75	437	0.93	AX-76383461	3835952	0.0448	0.711
pCO_2_20	28	4	4.49	328	0.89	AX-76385219	4167579	0.0239	0.706
pCO_2_28	9	20	0.59	462	0.94	AX-75706074	19358758	0.0070	0.416
pCO_2_28	1	110	0.54	194	0.38	AX-80866127	110487208	0.0098	0.510
pCO_2_28	27	2	0.53	650	0.96	AX-76356017	2038872	0.0065	0.653
pCO_2_28	10	3	0.50	447	0.91	AX-75607032	3037730	0.0069	0.626
pCO_2_28-20	28	4	0.61	328	0.83	AX-76384843	4097788	0.0076	0.296
pCO_2_28-20	23	2	0.57	388	0.86	AX-76282215	2594470	0.0071	0.435
pCO_2_28-20	3	14	0.56	287	0.81	AX-76421954	14679413	0.0075	0.323
pCO_2_28-20	10	1	0.50	393	0.86	AX-75601081	1816619	0.0080	0.339
pO_2_28	13	5	0.57	277	0.79	AX-75758019	5130673	0.0070	0.531
BE20	18	3	0.68	401	0.93	AX-75894740	3342614	0.0103	0.652
BE20	18	6	0.52	342	0.84	AX-75906711	6859485	0.0078	0.554
BE28	27	2	0.70	650	0.97	AX-76359325	2733806	0.0076	0.473
BE28	1	172	0.54	202	0.67	AX-75342016	172010216	0.0094	0.683
BE28	21	4	0.50	521	0.91	AX-76247040	4491122	0.0078	0.321
HCO_3_28	Z	30	4.11	74	0.47	AX-77209983	30284984	0.1864	0.671
HCO_3_28	Z	8	3.10	24	0.27	AX-80958477	8485438	0.0719	0.357
HCO_3_28	Z	5	2.22	62	0.33	AX-80834191	5042699	0.0931	0.634
HCO_3_28	Z	33	2.09	45	0.28	AX-80973925	33940034	0.0543	0.608
HCO_3_28	Z	35	1.67	128	0.50	AX-80901519	35319963	0.0589	0.379
HCO_3_28	Z	7	1.47	2	0.9	AX-77264084	7705768	0.0806	0.311
HCO_3_28	Z	70	1.45	55	0.28	AX-77257752	70210948	0.0625	0.376
HCO_3_28	Z	69	1.31	113	0.40	AX-80879264	69810199	0.0525	0.370
HCO_3_28	6	25	0.75	325	0.86	AX-76932184	25826439	0.0083	0.466
HCO_3_28	Z	71	0.74	183	0.62	AX-80943753	71554374	0.0520	0.360
HCO_3_28	6	26	0.53	291	0.83	AX-76933234	26203623	0.0092	0.493
TCO_2_28	Z	69	3.63	113	0.46	AX-80879264	69810199	0.1357	0.370
TCO_2_28	Z	30	3.04	74	0.44	AX-77209983	30284984	0.1424	0.671
TCO_2_28	Z	8	2.30	24	0.25	AX-80958477	8485438	0.0859	0.357
TCO_2_28	Z	33	1.73	45	0.28	AX-80973925	33940034	0.0667	0.608
TCO_2_28	Z	5	1.60	62	0.29	AX-80834191	5042699	0.0700	0.634
TCO_2_28	Z	7	1.23	2	0.7	AX-77264084	7705768	0.0707	0.311
TCO_2_28	Z	70	0.91	55	0.26	AX-77257752	70210948	0.0434	0.376
TCO_2_28	Z	35	0.80	128	0.48	AX-80901519	35319963	0.0419	0.379
TCO_2_28	6	25	0.66	325	0.85	AX-76932184	25826439	0.0080	0.466
TCO_2_28	26	3	0.51	616	0.98	AX-80958155	3785485	0.0079	0.513
TCO_2_28-20	10	5	0.51	515	0.93	AX-75615576	5758221	0.0067	0.355
K20	10	16	0.76	372	0.92	AX-75589587	16018566	0.0041	0.249
K20	10	18	0.53	496	0.96	AX-75597981	18294286	0.0038	0.278
K20	12	17	0.53	242	0.72	AX-75701199	17759131	0.0043	0.646
K20	26	3	0.50	616	0.96	AX-76340450	3273628	0.0036	0.180
K28-20	12	16	1.29	246	0.75	AX-75696568	16220734	0.0036	0.650
K28-20	12	17	0.69	242	0.70	AX-75701149	17743731	0.0043	0.633
iCa28	26	3	0.52	616	0.96	AX-76343628	3922118	0.0076	0.550
Hct28	14	11	1.78	391	0.94	AX-75776707	11791127	0.0096	0.502
Hct28	1	169	1.17	196	0.83	AX-75336362	169571235	0.0110	0.714
Hct28	28	3	1.01	437	0.92	AX-76384000	3944019	0.0116	0.397
Hct28	28	4	0.93	328	0.91	AX-76385356	4197143	0.0113	0.408
Hct28	22	3	0.88	573	0.95	AX-76269662	3474970	0.0072	0.513
Hct28	10	16	0.59	372	0.90	AX-75589730	16057907	0.0070	0.254
Hb28	14	11	1.64	391	0.95	AX-75776707	11791127	0.0091	0.502
Hb28	1	169	1.33	196	0.83	AX-75337336	169979876	0.0121	0.322
Hb28	28	3	0.96	437	0.91	AX-76384000	3944019	0.0112	0.397
Hb28	28	4	0.94	328	0.92	AX-76385356	4197143	0.0103	0.408
Hb28	22	3	0.79	573	0.95	AX-76269662	3474970	0.0073	0.513
Hb28	10	16	0.60	372	0.90	AX-75590148	16177101	0.0077	0.295
Hb28	1	170	0.50	176	0.65	AX-75337520	170074107	0.0083	0.676
Hb28-20	22	3	1.71	573	0.96	AX-76272400	3857927	0.0072	0.533
sO_2_28	25	0	1.23	364	0.91	AX-75758019	5130673	0.0070	0.531
sO_2_28	24	3	0.55	581	0.94	AX-76328225	36480	0.0111	0.618
sO_2_28-20	17	6	0.53	324	0.83	AX-75872796	6506736	0.0066	0.412
sO_2_28-20	17	7	0.53	467	0.88	AX-75875111	7125729	0.0066	0.172
Glu20	10	4	0.67	548	0.95	AX-80975590	4452892	0.0067	0.740
Glu28	22	3	1.09	573	0.94	AX-76273189	3966852	0.0070	0.585
Glu28	Z	5	0.79	62	0.28	AX-80834191	5042699	0.0246	0.634
Glu28	Z	70	0.74	55	0.24	AX-77257752	70210948	0.0266	0.376
Glu28	Z	69	0.64	113	0.42	AX-80879264	69810199	0.0187	0.371

^a^Blood chemistry components were measured pre-heat (day 20), on the seventh day of heat treatment (day 28), and the calculated differential due to heat (day 28–20)

^b^Frequency in which the window was included in the MCMC iterations (post-burn-in)

^c^SNP within the specified window which was most frequently included in the MCMC iterations (post-burn-in), and is therefore predicted to have the greatest effect on the phenotype

^d^Position of SNPs in base pairs on Gallus-gallus (version 4.0) chromosome

^e^Frequency in which the SNP was included in the MCMC iterations (post-burn-in) model

^f^Allele frequency of the SNP in the genotyped population (*N* = 458)

Six QTL for pH phenotypes were identified: three for pH20 with two on GGA18 and one on GGA28, one for pH28 on GGA12, and two for pH28-20 with one each on GGA6 and GGA10.

Nine QTL for pCO_2_ measurements were identified: one for pCO_2_20 on GGA28, four for pCO_2_28 located on GGA1, 9, 10, and 27, and four for pCO_2_28-20 on GGA3, 10, 23, and 28. No QTL were identified for pO_2_20 or for pO_2_28-20. One QTL was identified for pO_2_28 on GGA13.

A total of five QTL were identified for BE traits: two for BE20 on GGA18, three for BE28 with one each on GGA1, 21, and 27, and none for BE28-20. Nine QTL were identified for TCO_2_ traits: none for TCO_2_20; eight for TCO_2_28 one each on GGA6 and GGA26, and six on GGAZ, and one for TCO_2_28-20 on GGA10. No QTL were identified for HCO_3_20 or HCO_3_28-20, while seven were revealed for HCO_3_28 with one on GGA6 and six on GGAZ.

Five QTL for K traits were identified: four for K20 with two on GGA10, one on GGA12, and one on GGA26, none for K28 and one for K28-20 located on GGA12. No QTL were identified for the Na phenotypes. A single QTL was identified for ionized Ca phenotypes: for iCa28 on GGA26.

We identified five QTL for Hct measurements: none for Hct20 or Hct28-20, and five for Hct28 located one each on GGA1, 10, 14, 22, and two on GGA28. Seven QTL were identified for Hb: none for Hb20, six for Hb28 located one each on GGA1, 10, 14, 22, and two on 28, and one for Hb28-20 on GGA22. There were three QTL for sO_2_ phenotypes: none for sO_2_20, two for sO_2_28 located on GGA24 and GGA25, and one for sO_2_28-20 on GGA17.

Four QTL were identified for Glu: one for Glu20 on GGA10, and three for Glu28 with one on GGA22 and two on GGAZ.

### Pathway analysis

The pathway analysis of all annotated genes within significant QTL regions across all measured traits, and separately for genes in the regions of QTL co-localization, and the top 20 significant (*P* ≤ 0.05) canonical pathways for each group are listed in Table 3. Of the 999 genes identified within all significant QTL regions, 682 genes were annotated within IPA and used for the pathway analysis. Two canonical pathways of interest for all identified QTL include the AMPK signalling and Angiopoietin signalling pathways. Of the 226 genes in regions of QTL co-localization, 185 were annotated within IPA and used for pathway analysis. A pathway of particular interest that was revealed was the Cardiac Hypertrophy signalling pathway.

**Table 3:** Top 20 canonical pathways for QTL identified for all traits, and for co-localized QTL

Pathways for all identified QTL			
Pathway	*P*-value	Ratio:	Genes in pathway that were identified in current study
1D-myo-inositol Hexakisphosphate Biosynthesis II (Mammalian)	1.93E-03	4/19	INPP5E,IPMK,SEC16A,PMPCA
AMPK Signaling	2.15E-03	13/178	CHRNA5,MTOR,STRADA,AK8,INSR,CHRNA3,PPM1J,CHRNB4,PIK3R2,ADRA2A,TSC1,FOXO1,ADRA1A
Angiopoietin Signaling	1.22E-03	6/66	NRAS,PIK3R2,BIRC5,CASP9,IKBKAP,FOXO1
Calcium Signaling	1.51E-02	11/178	CALR,CHRNA5,MYL4,CHRNB4,CAMK4,CHRNA3,CAMK1G,MEF2D,TPM1,RAP1A,MEF2A
Cardiac Hypertrophy Signaling	5.80E-03	14/223	MTOR,MYL4,CAMK4,RHOC,IGF1R,NRAS,PIK3R2,RHOT1,ADRA2A,MEF2D,MAP3K3,CACNA1D,MEF2A,ADRA1A
D-myo-inositol (1,3,4)-trisphosphate Biosynthesis	1.93E-03	4/19	INPP5E,IPMK,SEC16A,PMPCA
D-myo-inositol (1,4,5)-trisphosphate Degradation	1.44E-02	3/18	INPP5E,SEC16A,PMPCA
Dopamine Degradation	8.29E-03	4/28	ALDH1A1,ALDH1A3,MAOB,ALDH4A1
ERK5 Signaling	2.28E-03	7/63	MAP2K5,NRAS,NTRK1,MEF2D,NGF,MAP3K3,MEF2A
Ethanol Degradation IV	4.02E-03	4/23	ALDH1A1,TYRP1,ALDH1A3,ALDH4A1
Glioblastoma Multiforme Signaling	1.03E-02	10/146	WNT2B,IGF1R,NRAS,MTOR,PIK3R2,WNT5A,RHOC,RHOT1,TSC1, FOXO1
Glioma Signaling	7.71E-03	8/98	ABL1,TGFA,IGF1R,NRAS,MTOR,PIK3R2,CAMK4,CAMK1G
Histamine Degradation	1.22E-02	3/17	ALDH1A1,ALDH1A3,ALDH4A1
Human Embryonic Stem Cell Pluripotency	1.85E-03	11/134	WNT2B,PIK3R2,WNT5A,SMAD3,SMAD6,NTRK1,TCF7L2,BMP2,NGF,FOXO1,NOG
Non-Small Cell Lung Cancer Signaling	1.13E-02	6/65	ABL1,TGFA,NRAS,PIK3R2,CASP9,RXRA
Nur77 Signaling in T Lymphocytes	1.26E-03	7/57	MAP2K5,SIN3B,CASP9,RXRA,CAMK4,MEF2D,MAP3K3
Putrescine Degradation III	2.84E-03	4/21	ALDH1A1,ALDH1A3,MAOB,ALDH4A1
Superpathway of D-myo-inositol (1,4,5)-trisphosphate Metabolism	4.71E-03	4/24	INPP5E,IPMK,SEC16A,PMPCA
Thyroid Cancer Signaling	9.69E-04	6/40	NRAS,RET,RXRA,NTRK1,TCF7L2,NGF
Tryptophan Degradation X (Mammalian, via Tryptamine)	4.02E-03	4/23	ALDH1A1,ALDH1A3,MAOB,ALDH4A1
Pathways identified for co-localized QTL			
Pathway	*P-value*	Ratio:	Genes in pathway that were identified in current study
2-oxobutanoate Degradation I	4.22E-02	1/5	MCEE
AMPK Signaling	4.42E-03	6/178	CHRNA5,PPM1J,CHRNB4,INSR,CHRNA3,ADRA1A
Calcium Signaling	1.55E-04	8/178	CALR,CHRNA5,CHRNB4,CHRNA3,CAMK1G,TPM1,RAP1A,MEF2A
Cardiac Hypertrophy Signaling	4.35E-02	5/223	IGF1R,NRAS,RHOC,MEF2A,ADRA1A
CDK5 Signaling	4.94E-02	3/105	NRAS,PPM1J,NGF
Cholecystokinin/Gastrin-mediated Signaling	4.95E-02	3/245	NRAS,RHOC,MEF2A
CTLA4 Signaling in Cytotoxic T Lymphocytes	4.01E-02	3/88	PPM1J,PTPN22,AP1M1
ERK5 Signaling	1.69E-02	3/63	NRAS,NGF,MEF2A
Germ Cell-Sertoli Cell Junction Signaling	4.93E-02	4/160	NRAS,TJP1,RHOC,RAB8B
Glioblastoma Multiforme Signaling	3.73E-02	4/146	WNT2B,IGF1R,NRAS,RHOC
Glioma Signaling	1.01E-02	4/98	TGFA,IGF1R,NRAS,CAMK1G
Integrin Signaling	3.33E-02	5/207	NRAS,TSPAN2,RHOC,TLN2,RAP1A
Methylmalonyl Pathway	3.39E-02	1/4	MCEE
mTOR Signaling	2.28E-02	5/187	NRAS,PPM1J,INSR,RHOC,RPS15
NF-κB Signaling	1.65E-02	5/172	TGFA,IGF1R,NRAS,INSR,NGF
PTEN Signaling	1.89E-02	4/118	IGF1R,NRAS,INSR,MAGI3
Renal Cell Carcinoma Signaling	2.32E-02	3/71	TGFA,NRAS,RAP1A
STAT3 Pathway	2.49E-02	3/73	IGF1R,NRAS,INSR
TCA Cycle II (Eukaryotic)	1.65E-02	2/23	IDH3A,ACO1
Thyroid Cancer Signaling	4.62E-02	2/40	NRAS,NGF

All characterized genes within significant QTL regions were used as input in Ingenuity Pathway Analysis (IPA) software. The Top 20 significant (*P* ≤ 0.05) pathways are listed. The results are displayed for pathways identified when using all QTL regions (61 total QTL) which resulted in 682 (999 total) annotated genes used for pathway analysis. The bottom section of the table displays the pathways identified when using only the co-localized QTL regions (7 total co-localized QTL regions) which resulted in 185 (226 total) annotated genes used for pathway analysis. The pathways are the top canonical pathways identified by IPA and are listed in alphabetical order. The ratio refers to the number of genes that were identified in the current study compared to the total number of genes that are in the pathway according to IPA

### Candidate genes

We explored regions of QTL co-localization in detail to identify candidate genes that may give insight into the complex biological mechanisms that control blood component response to heat stress. Candidate genes were identified using Ensemble Biomart within the 1 Mb windows that were significant for 3 or more traits (Additional file 1: Table S1).

## Discussion

The aim of this study was to identify and estimate the effect of QTL, and to perform a functional analysis using positional candidate genes, for blood components (pH, pCO_2_, pO_2_, base excess, HCO_3_, TCO_2_, K, Na, ionized Ca, Hct, Hb, sO_2_, and Glu) using a novel AIL of chickens under heat stress and a 600 K SNP panel for genotyping. The blood components measured were within the accepted range reported for chicken [19]. Blood chemistry components are grouped into functional categories (i.e., respiratory alkalosis, metabolic alkalosis, blood volume and oxygen carrying capacity, electrolytes, and glucose) for discussion.

### Population studied

Previous generations of this AIL were used for several QTL mapping studies and allowed the identification of many QTL including 257 for growth and body composition [20–24], 93 for skeletal integrity [25], 51 for metabolic traits [18], 12 for response to *Salmonella enteritidis* challenge [26–28], and 35 for response to heat stress [29]. Therefore, collectively, a wide range of traits have been associated with a large number of QTL in this AIL. The continued erosion of Linkage Disequilibrium (LD) in this population over subsequent generations, combined with the availability of larger SNP panels, creates a unique opportunity to more finely map the location of QTL that are in LD with a causal mutation.

### Respiratory alkalosis

#### Phenotypic measurements

During periods of intense heat, chickens increase the depth and frequency of respiration to decrease core body temperature [30]. Broilers that are heat stressed increase panting and display signs of respiratory alkalosis [10], which is caused by an increase in the amount of CO_2_ expelled from the lungs, and a consequent increase in pH within the blood, and an increase in pO_2_ within the blood. We investigated blood pH, pCO_2_, and pO_2_ to characterize respiratory alkalosis induced by heat stress.

Occurrence of respiratory alkalosis was clearly demonstrated in the current study by a significant increase in blood pH and significant decrease in pCO_2_ due to heat treatment, in agreement with previous studies. Heat stress for two hours at 32 °C in broilers at 35 days of age significantly increases blood pH and decreases pCO_2_ [31] and, in another study using broilers, heat stressed at 32 °C for 2 weeks at 28 days of age in birds that were panting [10]. We found pO_2_ increased in response to heat treatment, although not significantly. In a study using 35 day old broilers, blood pO_2_ significantly increased after cyclical heat stress for 10 days at 35 °C [32].

### Heritabilities

Only one other published study has estimated heritabilities of blood components in chickens under thermal stress [33]. The current study, therefore, adds substantially to the body of information on response of birds to thermal stress by estimating heritabilities of blood component levels and changes under heat stress and thermoneutral conditions. In broiler chickens at 22 days of age reared under cold stress conditions, heritabilities for blood pH, pCO_2_, and pO_2_ were estimated at 0.15, 0.15, and 0.03, respectively [33], in agreement with the current study’s estimates for thermoneutral and heat conditions. Our estimates for the changes in these blood components due to heat treatment was much lower, suggesting that the ability to select for the response to heat stress may be difficult.

### GWAS

To our knowledge, QTL for blood pH, pCO_2_, and pO_2_ in chickens have not been previously reported. Identification of QTL for blood pH on different chromosomes across measurement phases, indicates that genetic control of these traits exists and is partly dependent on the environment. Co-localized QTL for pCO_2_20 and pCO_2_28-20 on GGA28, and for pCO_2_28 and pCO_2_28-20 on GGA10, suggest that the same genetic regions contribute to control of pCO_2_ level independent of environmental temperature. The presence of co-localized QTL between measurement phases was not expected, based on the lack of phenotypic correlations (*r* = 0.00).

### Metabolic alkalosis

#### Phenotypic measurements

Metabolic alkalosis occurs when there is a disturbance in the fixed acids and bases in the extracellular fluid [11]. Imbalance of dietary Na, K, or Ca can result in metabolic alkalosis [34], which is characterized by an increase in blood pH, HCO_3_, and base excess, and can be induced in growing layers by high levels of calcium in feed [35].

Base excess is considered a comprehensive measure of the metabolic components of bases, which reflects the nonrespiratory contribution to changes in acid–base disturbances [36]. Base excess can be altered by changing the cation:anion ratio in the diet of broiler chickens and is associated with body weight and bone density [37]. In the current study, base excess significantly increased after heat treatment, which is consistent with the hypothesis that chickens experience metabolic alkalosis under heat stress.

HCO_3_ is the most abundant buffer in the blood, is primarily regulated by the kidneys, and is a metabolic component of acid–base balance [36]. We observed a significant increase in HCO_3_ due to heat treatment. These results contrasted with a previous study using broilers at 28 days of age in which blood HCO_3_ significantly decreased in panting birds under acute heat stress [10], and another study using male broilers that reported a decrease in HCO_3_ after a heat stress at 32 °C for 10 h [13]. TCO_2_ also increased in response to heat treatment. It was unexpected to observe a decrease in base excess, consistent with metabolic alkalosis, while HCO_3_ and TCO_2_ increased, because the traits are highly positively correlated within all treatment phases (*r* ≥ 0.95).

### Heritabilities

We estimated heritability of base excess between 0.00-0.10, of HCO_3_ between 0.03-0.23, and of TCO_2_ between 0.01-0.13. In broiler chickens at 22 days of age reared under cold stress conditions, blood HCO_3_ and TCO_2_ heritability were both estimated at 0.19 [33].

### GWAS

We are the first to report QTL in chickens for blood base excess, HCO_3_, and TCO_2_, which are related to metabolic alkalosis. QTL for base excess are located on separate chromosomes for all measurement phases, indicating a strong genetics by environmental (G x E) temperature interaction. The phenotypic correlations for base excess between measurement phases were both very low (*r* = 0.03). The QTL for base excess on GGA18 overlap with pH measured at thermoneutrality and were highly correlated (*r* = 0.78). Surprisingly, QTL for HCO_3_ were only identified during heat treatment and were on GGA6 and GGAZ. Ten of the eleven QTL for TCO_2_ measured during heat co-localized with QTL for HCO_3_ and these co-localized regions were located on GGA6, 26, and Z.

### Electrolytes

#### Phenotypic measurements

Blood K and Na levels numerically increased and iCa statistically increased in response to heat treatment. This is in disagreement with previous reports of decreasing levels of both K and Na in response to heat stress, likely due to increased water intake which results in decreased concentrations of electrolytes within the blood [6, 13, 38].

### Heritabilities

Heritability of K and Na blood levels in humans has been estimated to be very low, 0.03 and 0.04, respectively [39], in agreement with our low heritability estimates during heat and for the calculated differential. In contrast, our estimates for heritability under thermoneutral conditions for K and Na were higher, 0.20 and 0.08, respectively. Estimated heritability was 0.02 for ionized Ca measured during heat stress, lower than the 0.19 of mice in thermoneutral conditions [40]. The estimated heritability was low, for both thermoneutral (0.04) and the differential due to heat (0.01), indicating the genetic component for ionized Ca is dependent upon environmental conditions at the time of measurement. The low heritabilities of these traits during heat and for the calculated differential due to heat treatment suggest it may be difficult to select for these traits.

### GWAS

This research is the first to describe QTL for the electrolyte-balance traits of blood K, Na, and ionized Ca in the chicken. In swine, QTL have been identified for these traits [41]. QTL for blood K were located on GGA10, 12, and 26. QTL were identified for K across the thermoneutral and differential due to heat measurement phases, indicating genetic control of this component in this region on GGA12 despite environmental temperature. The correlation between thermoneutral and the differential was moderate (*r* = 0.10). No significant QTL for Na were identified in the current study and a single QTL for ionized Ca was located on GGA26 for the measurement taken during heat.

### Blood volume and oxygen saturation

#### Phenotypic measurements

Changes in blood volume and oxygen carrying capacity occur in chickens during periods of heat stress [5]. Both hematocrit and hemoglobin significantly increased due to heat treatment, which may be the result of dehydration. This result contrasts with a previous study using male broilers in which both decreased after an acute heat stress at 32 °C for 10 h [6]. Blood sO_2_ is a measure of oxyhemoglobin in relation to total hemoglobin that is able to bind oxygen [36], and this significantly increased during heat treatment.

### Heritability

The heritability of Hct was estimated as very low at 0.01 and 0.02 for pre-heat and the differential, respectively, while during heat was moderately heritable at 0.21. Heritability has been estimated for hematocrit at 0.39 in domestic fowl [42]. The increase in heritability when measured during heat stress indicates that this trait may be useful for selection. Heritability estimates of sO_2_ were very low (0.01-0.03), which is in general agreement with a previously reported value of 0.07 in cold-stressed broiler chickens at 22 days of age [33].

### GWAS

Seven QTL for haematocrit have been identified in chickens (www.animalgenome.org). In a broiler by layer F2 intercross, QTL for hematocrit were located on GGA1, 2, 6, and 14 [43]; in a Fayoumi by Leghorn F2 intercross on GGA1 and GGA15 [44], and in a broiler by layer cross on GGA1 [45]. Our current work confirmed previously identified QTL for Hct28 on GGA1 and GGA14. Novel QTL for Hct were on GGA10, 22, and 28. Most of the QTL identified in the current study for Hb co-localized with those identified for Hct, with the addition of a relatively large QTL for Hb28-20 on GGA22, explaining 1.7 % of the genetic variation. The co-localization of QTL among Hct and Hb is expected because they have very high positive phenotypic correlations across all measurement phases (*r* ≥ 0.99). We identified novel QTL for sO_2_ on GGA17, 24, and 25, none of which overlapped between measurement phases, indicating separate genetic control of this trait dependent upon environmental temperature. A previous study using a commercial broiler line identified one on GGA16 [46]. Thus, QTL for sO_2_ appear to be population specific.

### Glucose

#### Phenotypic measurement

Glucose is the body’s primary source of energy, and blood Glu significantly decreased due to heat treatment in the current study. In contrast, male broilers had a significant increase in Glu after heat stress at 32 °C for 10 h [6], and in broiler chicks of 5 weeks of age at 35-40 °C [47]. In chicken lines divergently selected for blood glucose concentration, the low glucose line was less efficient at food utilization compared to the high glucose line [48], which may indicate that the decrease in glucose we see during heat stress may contribute to inefficiency in food utilization.

### Heritability

The current study estimated heritabilities for glucose ranging between 0.02-0.19. In a study using chickens divergently selected for blood glucose concentration, heritability was estimated at 0.25 [48].

### GWAS

We identified QTL for Glu20 and Glu28 on GGA10, 22, and Z, while QTL were mapped to GGA2, 7, and Z in the F2 generation of the same chicken population under thermoneutral conditions [18]. The two studies may have detected the same QTL on chromosome Z and, due to the breakdown of LD over the generations, the current study may have mapped the QTL more accurately. In an F2 intercross between fat and lean broilers, QTL were identified for blood glucose on GGA3 and GGA18 [49], and for fasting plasma glucose on GGA5, 6, 13, and 26 [15]. A study using an F2 of broilers divergently selected for growth, identified QTL for plasma glucose on GGA20 and GGA27 [16]. Thus, QTL location for blood glucose level appears to be heat and/or population specific.

### Pathway analysis

Considering all measured traits, we identified a total of 32 unique QTL. All annotated genes within the QTL regions were used for pathway analysis using IPA and many significantly associated canonical pathways were identified including AMPK signalling and Angiopoietin signalling were identified. The top 20 pathways are found in Table 3. AMPK is a master metabolic regulator involved in metabolism [50] and, thus, may be a pathway which warrants further investigation for involvement in production traits during heat stress. During high ambient temperatures chickens redirect blood flow to the body surface to decrease body temperature [5], and the angiopoietin signalling pathway functions in blood vessel development which may help alleviate temperature stress.

The co-localized regions resulted in many significant canonical pathways and the top 20 pathways are found Table 3. Of particular interest is the Cardiac Hypertrophy signalling pathway (*P* = 4.35E-02). QTL for hemoglobin and hematocrit represent 3 (7 total) regions of co-localization and there is a positive linear relationship between hematocrit and heart weight in chickens under heat stress [5]; therefore, this pathway likely contributes to the response to heat stress in chickens.

### Candidate genes for co-localized QTL

The QTL regions that co-localized for three or more traits were further investigated for positional, functional candidate genes to give further insight into the biological mechanisms involved in the response of blood components to heat stress. The identified genes are located in Additional file 1: Table S1.

There are 51 genes in the region on GGA10 between 3–6 Mb that contained QTL for Glu20, pCO_2_28, and TCO_2_28-20. With 2 of these 3 traits associated with CO_2_ concentration, *CA12* (carbonic anhydrase) is a likely candidate gene involved in the CO_2_ response to heat stress. Carbonic anhydrases catalyse the reaction of CO_2_ and H_2_O to form HCO_3_ and H+, and thus may stabilize blood acid base balance during heat stress. Another strong functional candidate in this region is *HSP40*, a member of the heat shock protein family that functions as a molecular chaperone to prevent cellular damage during heat stress [51]. A candidate gene in this region for glucose level is *GCNT3*, a glucosamine acetyl transferase which is associated with glucose metabolism in humans [52].

Fourteen genes were identified on GGA10 between 16–17 Mb, where QTLs co-localized for pH28-20, Hct28, Hb28, and K20. Many QTL in chicken have been identified in this region including those related to growth [22, 53–55], abdominal fat [23, 49, 56], and the stress-associated trait of fear response [57]. A strong candidate gene is *ALDH6* (aldehyde dehydrogenase) which functions to convert aldehydes to carboxylic acids. This gene may function to maintain blood acid base balance during heat stress. Another gene in this region is IGF1 (insulin like growth factor 1), which has many roles and is a biomarker for growth [58].

Four genes were identified on GGA22 between 3–4 Mbs, where QTL were co-localized for Hct28, Hb28, Hb28-20, and Glu28. To our knowledge, no QTL have been reported in this region. Because all traits were measured during heat treatment or as the differential, we propose these to be heat specific QTL. Candidate genes *TGFA* (pretransforming growth factor) and *ADRA1A* (adrenergic receptor) both regulate cell growth. It is known that metabolic changes occur during periods of heat in chickens that contribute to reduction in growth, independent upon feed intake [9].

There are 48 genes in the 1 Mb region on GGA26 between 3–4 Mbs, where QTL co-localized for TCO_2_28, K20, and iCa28. Notably, a QTL for tibia bone mineral density identified in a commercial broiler and layer cross is located within this region [59]. This co-localization suggests that this locus might be involved in both blood calcium and bone density, and therefore, may be an ideal candidate for further investigation to understand the physiological response to heat stress on bone mineral density.

There are 86 genes in the 2 Mb region on GGA28 between 3–5 Mb where QTLs co-localize for pH20, Hb28, Hct28, pCO_2_20, and pCO_2_28-20. A QTL for heart weight, relating to susceptibility of pulmonary hypertension [60] co-localizes with those identified here. Many of these genes are related to membrane transport of solutes and DNA transcription. The solute carriers *SLC39A3*, *SLC25A42* and *SLC35E1* were identified, as well as *CHERP* and *CIB3*, involved in calcium homeostasis. Transcription-related genes include *SUGP1*, which is involved in RNA splicing; *RFXANK*, a DNA-binding protein; *NR2C2AP*, a nuclear receptor protein; *DDX49*, an RNA helicase; *ELL*, an RNA polymerase II elongation factor; and *SIN3B* a transcriptional regulator.

On GGAZ, 2 genes were identified between 5–7 Mbs, where QTL co-localize for Glu28, HCO_3_28, and TCO_2_28. The only reported QTL near this region is for antibody response to KLH antigen [61]. Heat stress is known to reduce antibody titre in chickens [62], and this locus may be involved in the complex interaction of heat and antibody titre. Although, antibody levels were not measured in the current study. During periods of heat stress, DNA transcription, RNA translation, and cellular proliferation are altered [63] and we observed several genes in this region related to these particular responses including: *KIAA1328*, involved in chromosomal integrity during mitosis; and *TPGS2*, involved in tubulin formation.

On GGAZ, 21 genes were identified between 69–71 Mbs, where QTL co-localize for Glu28, HCO_3_28, and TCO_2_28. The one QTL that is near this region was identified in a previous generation of the same AIL as the current study, and is for bone mineral density [25]. A recent study found that heat stress in broilers results in decreased bone mineral density [64]. In humans, low serum bicarbonate levels are associated with decreased bone mineral density [65]. Although this relationship has yet to be elucidated in the chicken, further studies should investigate the association between blood chemistry variables and bone mineral density. The genes identified in the current study that are primarily involved in DNA transcription include *XPA*, which is a DNA repair protein, *FOXE3* which is part of the forkhead box, and *SNORA66* which is small nuclear RNA. Additionally, microRNAs *gga*-*mir*-*2954*, *gga*-*mir*-*2131*, and *gga*-*mir*-*1583* were identified in this region. An additional gene of interest identified was *DNAJA1*, which is part of the heat shock family of proteins.

### QTL for blood components reveal orthologous genes between chicken and swine

**Fig. 3:**
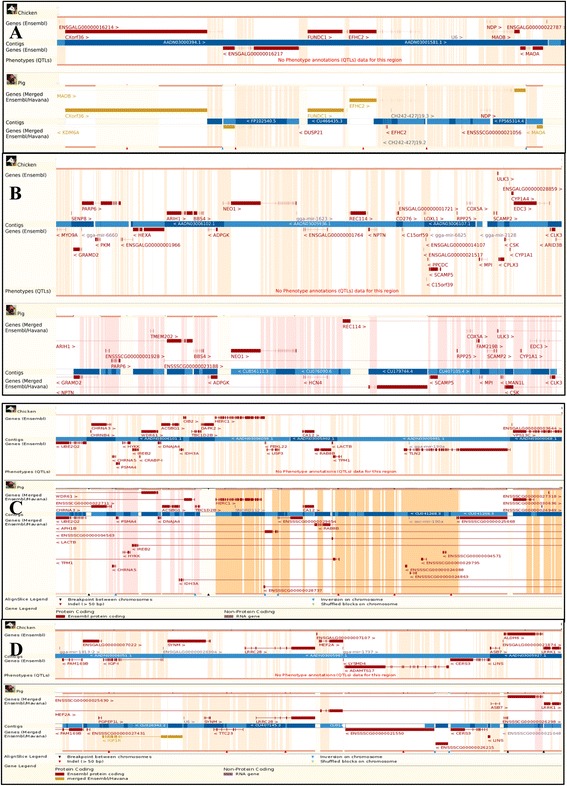
Syntenic regions between chicken and swine. Syntenic regions between chicken and pig containing QTL for blood component traits. **a** QTL for pCO_2_ in both chicken and pig. Chicken QTL on GGA1 at 110–111 Mb in chicken syntenic with pig on chromosome X, 43–44 Mb. **b**/**c** GGA10 1–2 Mb in chicken and pig chromosome 7 53–60 Mb **d**. GGA10 16–17 Mb and swine chromosome 1, 63–226 Mb

QTL for blood pCO_2_ in the current study were located on GGA1, 3, 9, 10, 23, 27, and 28. In swine, QTL for blood pCO_2_ are on chromosomes 6, 7, 8, 9, and X [41]. We identified a region of synteny between chicken GGA1, 110–111 Mb, and pig chromosome X, 43–44 Mb (Fig. 3a), which contains a pCO_2_ QTL and several orthologous genes including *FUNDC1*, *EFHC2*, *NDP*, and *MAOA*. Another region of synteny exists between chicken chromosome 10, 1–4 Mb, and pig chromosome 7, 53–65 Mb (Fig. 3b/c), which contains several orthologous genes including, but not limited to, *UBE2Q2*, *DNAJ*, *GRAMD2*, *ADPGK*, *NEO1*, *CLK3*, *SCAMP5*, *CSK*, and *MPI*. This region contains the carboxylic anhydrase gene (*CA12*) in chicken, which is involved in calcium metabolism, but this gene maps on pig chromosome 1, a chromosome on which no QTL have been reported for blood chemistry measurements. The region on GGA10, 1–4 Mb, contains QTL for Glu20, pCO_2_28, pCO_2_28-20, and TCO_2_28-20. The syntenic region in swine contains co-localized QTL for pCO_2_, HCO_3_, TCO_2_, and base excess [41].

A QTL for blood K level mapped to syntenic regions in chicken GGA10, 16–17 Mb, in our line and swine chromosome 1, 63–226 Mb (Fig. 3d) in a previous study [66]. An orthologous gene of interest in this region is *IGF*-*1*.

## Conclusions

The results of this study contribute to the currently sparse knowledge of levels and heritabilities of several blood components under thermoneutral and heat stress conditions in chickens. Most blood components changed in response to heat treatment. Mapped QTL may serve as markers for genomic selection to enhance heat tolerance in poultry and several candidate genes were identified which may give additional insight into mechanisms of physiologic response to high ambient temperatures.

## Methods

### Ethics statement

Animal experiments were approved by the Institutional Animal Care and Use Committee of Iowa State University: Log #4-11-7128-G.

### Chicken lines

We used the F18 and F19 generations of an AIL between chicken lines divergent for thermotolerence created by crossing a single broiler sire to six highly inbred Fayoumi dams [67]. Birds were reared in floor pens with wood shavings bedding and had ad libitum access to water and feed that met all NRC requirements [68].

### Heat stress experimental design

A total of 631 birds from four hatches (two hatches in each of the two generations) were used for independent heat stress experiments (four replicates). At 17 days of age, birds were transferred to environmentally controlled chambers and acclimated for five days. Multiple chambers, each containing 6 pens, were used per replicate. Ten to 12 birds were placed in each pen. From day 22 to 28 of age, the chambers heated to 35 °C for 7 h per day and remained at 25 °C at all other times.

### Blood variable measurements

Blood was collected from the wing vein on day 20 (pre-heat) and day 28 (during heat) using a heparinized syringe and needle, and analysed immediately using an iSTAT Portable Clinical Analyser [36]. The iSTAT CG8+ cartridge was utilized to measure thirteen blood variables including; pH, pCO_2_, pO_2_, base excess, HCO_3_, TCO_2_, K, Na, ionized Ca, hematocrit, hemoglobin, sO_2_, and glucose.

### DNA isolation and genotyping

Blood was collected from the wing vein by using an EDTA-coated syringe and needle, and stored at −20 °C. DNA was extracted using a salting out method. Briefly, whole blood was incubated with lysis buffer containing proteinase K. Proteins were precipitated out using 5 M NaCl while the supernatant remained. The supernatant was combined with 70 % ethanol to precipitate out DNA. The DNA isolated from 468 AIL, 6 broiler, and 6 Fayoumi chickens was genotyped on the Affymetrix 600 K chicken SNP axiom array [69] by GeneSeek Inc., Lincoln, NE. SNP chromosomal locations were based on the Gallus_gallus_4.0 assembly through Ensembl.

### Statistical analyses

Calculations of means and standard errors, fixed effects and covariates for the GWAS were calculated based on ANOVA (analysis of variance), and significant terms were fit as fixed effects with a *P* value ≤ 0.05 using JMP statistical software [70]. Heritabilities were estimated with an animal model using ASReml software [71].

Parameters for inclusion of SNP genotypes included SNP call rate ≥ 95 % and minor allele frequency ≥ 5 %. Genotyping console (Affymetrix) software was used to create genotyping calls and quality control based on whole animal DishQC score ≥ 0.7. The SNPolisher (Affymetrix) R package was used for quality control of individual SNP in all animals with passing DishQC scores.

The GWAS of phenotypic traits with SNP genotypes was done using GenSel software [72]. Bayes B, which fits all SNPs simultaneously as random effects, was used for the analysis. The mixed model used for the GWAS:

$$ y=Xb+{\displaystyle {\sum}_j^k{z}_j}{\alpha}_j{\delta}_j+\varepsilon . $$

Where **y** = vector form of phenotypes, **X** = incidence matrix to account for fixed effects on phenotypes, **b** = vector of fixed effects, **z**_**j**_ = vector of genotypes for SNP j based on the number of B alleles (−10, 0, +10, or the average of the genotypes at SNP j), α_**j**_ = allele substitution effect for SNP j, δ_**j**_ = whether SNP j was included in the Markov chain Monte Carlo (MCMC) chain, and ε is the error associated with the analysis.

The genomic markers were split into 1001 non-overlapping 1 Mb windows across the genome. A total of 41,000 MCMC iterations were run for each analysis and the first 1000 iterations were discarded (burn in). The δ_j_ was set so that π = 0.9978 to avoid fitting more SNPs than number of animals in a given iteration. In a true infinitesimal model, each window is expected to explain 0.1 % (100 %/1001) of the genetic variation; therefore, a 1 Mb window was considered significant if it explained ≥ 0.5 % of the total genetic variation, corresponding to 5 times more observed than expected.

### Pathway analysis

To further investigate QTL regions, we conducted a pathway analysis using Ingenuity Pathway Analysis (IPA) software. All annotated genes within significant (explaining ≥ 0.05 % of the genetic variation) 1 Mb windows for any measured trait were identified using Ensemble biomart. This gene list was used as input into IPA and a core analysis was completed using default parameters to identify significant (*P* ≤ 0.05) canonical pathways and the top 20 significant pathways were reported. Additionally, a gene list was created using the regions of QTL co-localization (3 or more traits) and analysed as described for all QTL regions.

### Candidate genes

Candidate genes were identified for regions of QTL co-localization (3 or more traits). All genes within the region were identified using ENSEMBL biomart [73].

### Syntenic regions between chicken and swine

To identify syntenic regions for reported QTL for the same blood chemistry component measurements between chicken and pig, the Comparative Genomics option was used in Ensembl [73].

### Availability of Data and Materials

The dataset supporting the conclusions of this article is available in the Animal QTLdb (animalgenome.org) repository and can be found at http://www.animalgenome.org/cgi-bin/QTLdb/GG/pubtails?PUBMED_ID=ISU0082. The phenotypic dataset supporting the conclusions of this article is included within the article as an additional file (Additional file 2: Table S2).

## Additional files

Additional file 1: Table S1. Positional candidate genes categorized by location for co-localized QTL. (DOCX 31 kb) Additional file 2: Phenotypic data for blood chemistry components in 468 advanced intercross line chickens. (XLSX 123 kb)

## Abbreviations

AIL: advanced intercross line
GWAS: genome wide association study
QTL: quantitative trait loci

## References

[1] USDA (2012). Climate Change Science White Paper.

[2] St-Pierre N, Cobanov B, Schnitkey G (2003). Economic losses from heat stress by US livestock industries. J Dairy Sci.

[3] Administration NOaA (1995). Summer of 1995 heat wave. Online.

[4] Wolfenson D, Frei YF, Snapir N, Berman A (1981). Heat stress effects on capillary blood flow and its redistribution in the laying hen. Pflugers Arch.

[5] Yahav S, Straschnow A, Plavnik I, Hurwitz S (1997). Blood system response of chickens to changes in environmental temperature. Poult Sci.

[6] Borges S, Da Silva AF, Majorka A, Hooge D, Cummings K (2004). Physiological responses of broiler chickens to heat stress and dietary electrolyte balance (sodium plus potassium minus chloride, milliequivalents per kilogram). Poult Sci.

[7] Garriga C, Hunter RR, Amat C, Planas JM, Mitchell MA, Moretó M (2006). Heat stress increases apical glucose transport in the chicken jejunum. Am J Physiol Regul Integr Comp Physiol.

[8] Austic R (1985). Feeding poultry in hot and cold climates.

[9] Lin H, Malheiros R, Moraes V, Careghi C, Decuypere E, Buyse J (2004). Acclimation of broiler chickens to chronic high environmental temperature. Archiv Fur Geflugelkunde.

[10] Teeter R, Smith M, Owens F, Arp S, Sangiah S, Breazile J (1985). Chronic heat stress and respiratory alkalosis: occurrence and treatment in broiler chicks. Poult Sci.

[11] Galla JH (2000). Metabolic alkalosis. J Am Soc Neph.

[12] Borges S, Fischer Da Silva A, Maiorka A (2007). Acid–base balance in broilers. World Poult Sci J.

[13] Borges S, Da Silva AF, Ariki J, Hooge D, Cummings K (2003). Dietary electrolyte balance for broiler chickens exposed to thermoneutral or heat-stress environments. Poult Sci.

[14] Schaal T, Arango J, Wolc A, Brady J, Fulton J, Rubinoff I (2015). Commercial Hy-Line W-36 pullet and laying hen venous blood gas and chemistry profiles utilizing the portable i-STAT® 1 analyzer. Poult Sci.

[15] Nadaf J, Pitel F, Gilbert H, Duclos MJ, Vignoles F, Beaumont C (2009). QTL for several metabolic traits map to loci controlling growth and body composition in an F2 intercross between high-and low-growth chicken lines. Physiol Gen.

[16] Park H-B, Jacobsson L, Wahlberg P, Siegel PB, Andersson L (2006). QTL analysis of body composition and metabolic traits in an intercross between chicken lines divergently selected for growth. Physiol Gen.

[17] Jacobsson L, Park H-B, Wahlberg P, Fredriksson R, Perez-Enciso M, Siegel PB (2005). Many QTLs with minor additive effects are associated with a large difference in growth between two selection lines in chickens. Genet Res.

[18] Zhou H, Evock-Clover C, McMurtry J, Ashwell C, Lamont S (2007). Genome-wide linkage analysis to identify chromosomal regions affecting phenotypic traits in the chicken. IV. Metabolic traits. Poult Sci.

[19] Martin MP, Wineland M, Barnes HJ (2010). Selected blood chemistry and gas reference ranges for broiler breeders using the i-STAT® handheld clinical analyzer. Avian Dis.

[20] Deeb N, Lamont S (2003). Use of a novel outbred by inbred F1 cross to detect genetic markers for growth. An Genet.

[21] Li H, Deeb N, Zhou H, Mitchell A, Ashwell C, Lamont S (2003). Chicken quantitative trait loci for growth and body composition associated with transforming growth factor-beta genes. Poult Sci.

[22] Zhou H, Deeb N, Evock-Clover C, Ashwell C, Lamont S (2006). Genome-wide linkage analysis to identify chromosomal regions affecting phenotypic traits in the chicken. I. Growth and average daily gain. Poult Sci.

[23] Zhou H, Deeb N, Evock-Clover C, Ashwell C, Lamont S (2006). Genome-wide linkage analysis to identify chromosomal regions affecting phenotypic traits in the chicken. II. Body composition. Poult Sci.

[24] Abasht B, Lamont S (2007). Genome-wide association analysis reveals cryptic alleles as an important factor in heterosis for fatness in chicken F2 population. An Genet.

[25] Zhou H, Deeb N, Evock-Clover C, Mitchell A, Ashwell C, Lamont S (2007). Genome-wide linkage analysis to identify chromosomal regions affecting phenotypic traits in the chicken. III. Skeletal integrity. Poult Sci.

[26] Kaiser M, Lamont S (2002). Microsatellites linked to Salmonella enterica Serovar Enteritidis burden in spleen and cecal content of young F1 broiler-cross chicks. Poult Sci.

[27] Kaiser M, Deeb N, Lamont S (2002). Microsatellite markers linked to Salmonella enterica serovar enteritidis vaccine response in young F1 broiler-cross chicks. Poult Sci.

[28] Liu W, Lamont S (2003). Candidate Gene Approach: Potentional Association of Caspase-1, Inhibitor of Apoptosis Protein-1, and Prosaposin Gene Polymorphisms with Response to Salmonella enteritidis Challenge or Vaccination in Young Chicks. An Biotech.

[29] Van Goor A, Bolek KJ, Ashwell CM, Persia ME, Rothschild MF, Schmidt CJ (2015). Identification of quantitative trait loci for body temperature, body weight, breast yield, and digestibility in an advanced intercross line of chickens under heat stress. Genet Sel Evol.

[30] Randall W, Hiestand W (1939). Panting and temperature regulation in the chicken. Am J Physiol.

[31] Sandercock D, Hunter R, Nute G, Mitchell M, Hocking P (2001). Acute heat stress-induced alterations in blood acid–base status and skeletal muscle membrane integrity in broiler chickens at two ages: Implications for meat quality. Poult Sci.

[32] Deyhim F, Teeter R (1991). Research note: sodium and potassium chloride drinking water supplementation effects on acid–base balance and plasma corticosterone in broilers reared in thermoneutral and heat-distressed environments. Poult Sci.

[33] Closter A, Van As P, Groenen M, Vereijken A, Van Arendonk J, Bovenhuis H (2009). Genetic and phenotypic relationships between blood gas parameters and ascites-related traits in broilers. Poult Sci.

[34] Daghir N (2008). Nutrient requirements of poultry at high temperature. Poultry production in hot climate.

[35] Guoa X, Huanga K, Chenga H, Luo J, Pana C (2008). High dietary calcium causes metabolic alkalosis in growing layers. Poult Sci.

[36] Chin CD, Linder V, Sia SK (2012). Commercialization of microfluidic point-of-care diagnostic devices. Lab Chip.

[37] Halley J, Nelson T, Kirby L, Johnson Z (1987). Effect of altering dietary mineral balance on growth, leg abnormalities, and blood base excess in broiler chicks. Poult Sci.

[38] Ait-Boulahsen A, Garlich J, Edens F (1989). Effect of fasting and acute heat stress on body temperature, blood acid–base and electrolyte status in chickens. Comp Biochem Physiol.

[39] Meyer TE, Verwoert GC, Hwang S-J, Glazer NL, Smith AV, Van Rooij F (2010). Genome-wide association studies of serum magnesium, potassium, and sodium concentrations identify six loci influencing serum magnesium levels. PLoS Genet.

[40] Tordoff MG, Bachmanov AA, Reed DR (2007). Forty mouse strain survey of voluntary calcium intake, blood calcium, and bone mineral content. Physiol Behav.

[41] Reiner G, Fischer R, Köhler F, Berge T, Hepp S, Willems H (2009). Heritabilities and quantitative trait loci for blood gases and blood pH in swine. An Genet.

[42] Washburn K (1967). Heritability of packed red blood cell volume in the domestic fowl. Poult Sci.

[43] Boschiero C, Jorge EC, Ninov K, Nones K, Do Rosário MF, Coutinho LL (2013). Association of IGF1 and KDM5A polymorphisms with performance, fatness and carcass traits in chickens. J Appl Genet.

[44] Der Laan M-H P-v, Bed’Hom B, Coville J-L, Pitel F, Feve K, Leroux S (2009). Microsatellite mapping of QTLs affecting resistance to coccidiosis (Eimeria tenella) in a Fayoumi × White Leghorn cross. BMC Genomics.

[45] Navarro P, Visscher P, Knott S, Burt D, Hocking P, Haley C (2005). Mapping of quantitative trait loci affecting organ weights and blood variables in a broiler layer cross. Brit Poult Sci.

[46] Ewald S, Ye X, Avendano S, McLeod S, Lamont S, Dekkers J (2007). Associations of BF2 alleles with antibody titres and production traits in commercial pure line broiler chickens. An Genet.

[47] Khan W, Khan A, Anjuman A, Rehman Z (2002). Effects of induced heat stress on some biochemical values in broiler chicks. Int J Agric Biol.

[48] Leclercq B, Simon J, Ricard F (1987). Effects of selection for high and low plasma glucose concentration in chickens. Brit Poult Sci.

[49] Demeure O, Duclos MJ, Bacciu N, Le Mignon G, Filangi O, Pitel F (2013). Genome-wide interval mapping using SNPs identifies new QTL for growth, body composition and several physiological variables in an F2 intercross between fat and lean chicken lines. Genet Sel Evol.

[50] Mihaylova MM, Shaw RJ (2011). The AMPK signalling pathway coordinates cell growth, autophagy and metabolism. Nat Cell Biol.

[51] Glover JR, Lindquist S (1998). Hsp104, Hsp70, and Hsp40: a novel chaperone system that rescues previously aggregated proteins. Cell.

[52] Dostrovsky N, Towheed T, Hudson R, Anastassiades T (2011). The effect of glucosamine on glucose metabolism in humans: a systematic review of the literature. Osteoarth Cart.

[53] Nassar M, Goraga Z, Brockmann G. Quantitative trait loci segregating in crosses between New Hampshire and White Leghorn chicken lines: IV. Growth performance. An Genet. 2015. 10.1111/age.12298 25908024

[54] Ambo M, Moura A, Ledur M, Pinto L, Baron E, Ruy D (2009). Quantitative trait loci for performance traits in a broiler × layer cross. An Genet.

[55] Zhou Y, Liu Y, Jiang X, Du H, Li X, Zhu Q (2010). Polymorphism of chicken myocyte-specific enhancer-binding factor 2A gene and its association with chicken carcass traits. Molec Biol Rep.

[56] Campos R, Nones K, Ledur M, Moura A, Pinto L, Ambo M (2009). Quantitative trait loci associated with fatness in a broiler–layer cross. An Genet.

[57] Buitenhuis A, Rodenburg T, Siwek M, Cornelissen S, Nieuwland M, Crooijmans R (2004). Identification of QTLs involved in open-field behavior in young and adult laying hens. Behav Genet.

[58] Renehan AG, Zwahlen M, Minder CT, O’Dwyer S, Shalet SM, Egger M (2004). Insulin-like growth factor (IGF)-I, IGF binding protein-3, and cancer risk: systematic review and meta-regression analysis. Lancet.

[59] Schreiweis M, Hester P, Settar P, Moody D (2006). Identification of quantitative trait loci associated with egg quality, egg production, and body weight in an F2 resource population of chickens1. An Genet.

[60] Rabie T, Crooijmans R, Bovenhuis H, Vereijken A, Veenendaal T, Poel JD (2005). Genetic mapping of quantitative trait loci affecting susceptibility in chicken to develop pulmonary hypertension syndrome. An Genet.

[61] Sun Y, Biscarini F, Bovenhuis H, Parmentier H, Poel VDJ (2013). Genetic parameters and across‐line SNP associations differ for natural antibody isotypes IgM and IgG in laying hens. An Genet.

[62] Mashaly M, Hendricks G, Kalama M, Gehad A, Abbas A, Patterson P (2004). Effect of heat stress on production parameters and immune responses of commercial laying hens. Poult Sci.

[63] Kültz D (2005). Molecular and evolutionary basis of the cellular stress response. Annu Rev Physiol.

[64] Hosseini-Vashan S, Golian A, Yaghobfar A (2015). Growth, immune, antioxidant, and bone responses of heat stress-exposed broilers fed diets supplemented with tomato pomace. Int J Biomet.

[65] Chen W, Melamed ML, Abramowitz MK (2015). Serum bicarbonate and bone mineral density in US adults. Am J Kid Dis.

[66] Reiner G, Clemens N, Fischer R, Köhler F, Berge T, Hepp S (2009). Mapping of quantitative trait loci for clinical–chemical traits in swine. An Genet.

[67] Deeb N, Lamont S (2002). Genetic architecture of growth and body composition in unique chicken populations. J Hered.

[68] NRC. Nut Requ Poult (1994). National Research Council.

[69] Kranis A, Gheyas AA, Boschiero C, Turner F, Yu L, Smith S (2013). Development of a high density 600 K SNP genotyping array for chicken. BMC Genomics.

[70] Institute S (2000). JMP statistical discovery software.

[71] Gilmour AR, Gogel B, Cullis B, Thompson R, Butler D (2009). ASReml user guide release 3.0. VSN Int Ltd.

[72] Fernando R, Garrick D (2008). GenSel user manual for a portfolio of genomic selection related analyses.

[73] Cunningham F, Amode MR, Barrell D, Beal K, Billis K, Brent S (2015). Ensembl 2015. Nucleic Acids Res.

