# Supplementary material for: Quantitative trait loci identified for blood chemistry components of an advanced intercross line of chickens under heat stress
Source: BMC Genomics. 2016 Apr 14;17:287. doi: 10.1186/s12864-016-2601-x (PMC4831167; doi:10.1186/s12864-016-2601-x)
Supplement: Additional file 1: Table S1. — Positional candidate genes categorized by location for co-localized QTL. (DOCX 31 kb) [file 12864_2016_2601_MOESM1_ESM.docx]

**Additional file 1: Table S1. Positional candidate genes categorized by location for co-localized QTL**

| **Position and traits** | **Gene name** | **Description** |
| --- | --- | --- |
| **GGA10: 3-4 Mb**  Glu20  pCO_2_28  TCO_2_28-20 | UBE2Q2 | ubiquitin-conjugating enzyme E2Q family member 2 [Source:HGNC Symbol;Acc:HGNC:19248] |
|  | CHRNB4 | neuronal acetylcholine receptor subunit beta-4 precursor [Source:RefSeq peptide;Acc:NP_990150] |
|  | CHRNA3 | Neuronal acetylcholine receptor subunit alpha-3 [Source:UniProtKB/Swiss-Prot;Acc:P09481] |
|  | CHRNA5 | neuronal acetylcholine receptor subunit alpha-5 precursor [Source:RefSeq peptide;Acc:NP_989746] |
|  | PSMA4 | proteasome subunit alpha type-4 [Source:RefSeq peptide;Acc:NP_001185531] |
|  | HYKK | aminoglycoside phosphotransferase domain-containing protein 1 [Source:RefSeq peptide;Acc:NP_001264388] |
|  | IREB2 | iron-responsive element-binding protein 2 [Source:RefSeq peptide;Acc:NP_001026625] |
|  | CRABP-I | Cellular retinoic acid-binding protein 1 [Source:UniProtKB/Swiss-Prot;Acc:P40220] |
|  | WDR61 | WD repeat-containing protein 61 [Source:RefSeq peptide;Acc:NP_001005805] |
|  | DNAJA4 | DnaJ (Hsp40) homolog, subfamily A, member 4 [Source:HGNC Symbol;Acc:HGNC:14885] |
|  | ACSBG1 | acyl-CoA synthetase bubblegum family member 1 [Source:HGNC Symbol;Acc:HGNC:29567] |
|  | IDH3A | isocitrate dehydrogenase [Source:RefSeq peptide;Acc:NP_001005808] |
|  | CIB2 | calcium and integrin binding family member 2 [Source:HGNC Symbol;Acc:HGNC:24579] |
|  | TBC1D2B | TBC1 domain family, member 2B [Source:HGNC Symbol;Acc:HGNC:29183] |
|  | DAPK2 | death-associated protein kinase 2 [Source:HGNC Symbol;Acc:HGNC:2675] |
|  | HERC1 | HECT and RLD domain containing E3 ubiquitin protein ligase family member 1 [Source:HGNC Symbol;Acc:HGNC:4867] |
|  | FBXL22 | F-box and leucine-rich repeat protein 22 [Source:HGNC Symbol;Acc:HGNC:27537] |
|  | USP3 | ubiquitin specific peptidase 3 [Source:HGNC Symbol;Acc:HGNC:12626] |
|  | CA12 | carbonic anhydrase XII [Source:HGNC Symbol;Acc:HGNC:1371] |
|  | RAB8B | RAB8B, member RAS oncogene family [Source:HGNC Symbol;Acc:HGNC:30273] |
|  | LACTB | serine beta-lactamase-like protein LACTB, mitochondrial [Source:RefSeq peptide;Acc:NP_001025717] |
|  | TPM1 | Tropomyosin alpha-1 chain [Source:UniProtKB/Swiss-Prot;Acc:P04268] |
|  | TLN2 | talin 2 [Source:HGNC Symbol;Acc:HGNC:15447] |
|  | VPS13C | vacuolar protein sorting 13 homolog C (S. cerevisiae) [Source:HGNC Symbol;Acc:HGNC:23594] |
|  | RORalpha1 | Uncharacterized protein [Source:UniProtKB/TrEMBL;Acc:F1NML9] |
|  | ICE2 | interactor of little elongation complex ELL subunit 2 [Source:HGNC Symbol;Acc:HGNC:29885] |
|  | Protein | Annexin A2 [Source:UniProtKB/Swiss-Prot;Acc:P17785] |
|  | BNIP2 | BCL2/adenovirus E1B 19kDa interacting protein 2 [Source:HGNC Symbol;Acc:HGNC:1083] |
|  | GTF2A2 | transcription initiation factor IIA subunit 2 [Source:RefSeq peptide;Acc:NP_001075177] |
|  | OTUD7A | OTU deubiquitinase 7A [Source:HGNC Symbol;Acc:HGNC:20718] |
|  | KLF13 | Kruppel-like factor 13 [Source:HGNC Symbol;Acc:HGNC:13672] |
|  | TRPM1 | transient receptor potential cation channel, subfamily M, member 1 [Source:HGNC Symbol;Acc:HGNC:7146] |
|  | MTMR10 | myotubularin related protein 10 [Source:HGNC Symbol;Acc:HGNC:25999] |
|  | FAN1 | FANCD2/FANCI-associated nuclease 1 [Source:HGNC Symbol;Acc:HGNC:29170] |
|  | MPHOSPH10 | M-phase phosphoprotein 10 (U3 small nucleolar ribonucleoprotein) [Source:HGNC Symbol;Acc:HGNC:7213] |
|  | MCEE | methylmalonyl CoA epimerase [Source:HGNC Symbol;Acc:HGNC:16732] |
|  | APBA2 | amyloid beta (A4) precursor protein-binding, family A, member 2 [Source:HGNC Symbol;Acc:HGNC:579] |
|  | FAM189A1 | family with sequence similarity 189, member A1 [Source:HGNC Symbol;Acc:HGNC:29075] |
|  | TJP1 | tight junction protein 1 [Source:HGNC Symbol;Acc:HGNC:11827] |
|  | TARSL2 | threonyl-tRNA synthetase-like 2 [Source:HGNC Symbol;Acc:HGNC:24728] |
|  | TM2D3 | TM2 domain containing 3 [Source:HGNC Symbol;Acc:HGNC:24128] |
|  | ADAL | adenosine deaminase-like protein [Source:RefSeq peptide;Acc:NP_001025718] |
|  | LARP6 | La ribonucleoprotein domain family, member 6 [Source:HGNC Symbol;Acc:HGNC:24012] |
|  | LRRC49 | leucine rich repeat containing 49 [Source:HGNC Symbol;Acc:HGNC:25965] |
|  | THSD4 | thrombospondin, type I, domain containing 4 [Source:HGNC Symbol;Acc:HGNC:25835] |
|  | FOXB1 | forkhead box B1 [Source:HGNC Symbol;Acc:HGNC:3799] |
|  | GCNT3 | glucosaminyl (N-acetyl) transferase 3, mucin type [Source:HGNC Symbol;Acc:HGNC:4205] |
|  | gga-mir-204-2 | gga-mir-204-2 [Source:miRBase;Acc:MI0001225] |
|  | gga-mir-1574 | gga-mir-1574 [Source:miRBase;Acc:MI0007300] |
|  | gga-mir-190a | gga-mir-190a [Source:miRBase;Acc:MI0001224] |
|  | uc_338 | TUC338 [Source:RFAM;Acc:RF02271] |
| GGA10: 16-17 Mb  pH28-20  Hct28  Hb28  K20 | FAM169B | family with sequence similarity 169, member B [Source:HGNC Symbol;Acc:HGNC:26835] |
|  | IGF-I | insulin-like growth factor 1 receptor precursor [Source:RefSeq peptide;Acc:NP_990363] |
|  | SYNM | synemin [Source:RefSeq peptide;Acc:NP_990140] |
|  | LRRC28 | leucine-rich repeat-containing protein 28 [Source:RefSeq peptide;Acc:NP_001005822] |
|  | MEF2A | myocyte-specific enhancer factor 2A [Source:RefSeq peptide;Acc:NP_990195] |
|  | LYSMD4 | LysM, putative peptidoglycan-binding, domain containing 4 [Source:HGNC Symbol;Acc:HGNC:26571] |
|  | ADAMTS17 | ADAM metallopeptidase with thrombospondin type 1 motif, 17 [Source:HGNC Symbol;Acc:HGNC:17109] |
|  | CERS3 | ceramide synthase 3 [Source:HGNC Symbol;Acc:HGNC:23752] |
|  | LINS1 | lines homolog 1 [Source:HGNC Symbol;Acc:HGNC:30922] |
|  | ASB7 | ankyrin repeat and SOCS box protein 7 [Source:RefSeq peptide;Acc:NP_001012788] |
|  | ALDH6 | aldehyde dehydrogenase family 1 member A3 [Source:RefSeq peptide;Acc:NP_990000] |
|  | LRRK1 | leucine-rich repeat kinase 1 [Source:HGNC Symbol;Acc:HGNC:18608] |
|  | gga-mir-1813-2 | gga-mir-1813-2 [Source:miRBase;Acc:MI0007443] |
|  | gga-mir-1737 | gga-mir-1737 [Source:miRBase;Acc:MI0007476] |
| GGA22: 3-4 Mb  Hct28  Hb28  Hb28-20 | TGFA | protransforming growth factor alpha precursor [Source:RefSeq peptide;Acc:NP_001001614] |
|  | ADRA1A | adrenoceptor alpha 1A [Source:HGNC Symbol;Acc:HGNC:277] |
|  | ANXA4 | annexin A4 [Source:HGNC Symbol;Acc:HGNC:542] |
|  | LRRTM4 | leucine rich repeat transmembrane neuronal 4 [Source:HGNC Symbol;Acc:HGNC:19411] |
| GGA26: 3-4 Mb  TCO_2_28  K20  iCa28 | CAMK1G | calcium/calmodulin-dependent protein kinase IG [Source:HGNC Symbol;Acc:HGNC:14585] |
|  | LAMB3 | laminin, beta 3 [Source:HGNC Symbol;Acc:HGNC:6490] |
|  | G0S2 | G0/G1switch 2 [Source:RefSeq peptide;Acc:NP_001177853] |
|  | TRAF3IP3 | TRAF3 interacting protein 3 [Source:HGNC Symbol;Acc:HGNC:30766] |
|  | IRF6 | interferon regulatory factor 6 [Source:HGNC Symbol;Acc:HGNC:6121] |
|  | GUCA1B | Guanylyl cyclase-activating protein 2 [Source:UniProtKB/Swiss-Prot;Acc:P79881] |
|  | GUCA1A | Guanylyl cyclase-activating protein 1 [Source:UniProtKB/Swiss-Prot;Acc:P79880] |
|  | C6orf132 | chromosome 6 open reading frame 132 [Source:HGNC Symbol;Acc:HGNC:21288] |
|  | WDR77 | methylosome protein 50 [Source:RefSeq peptide;Acc:NP_001026087] |
|  | ATP5F1 | ATP synthase, H+ transporting, mitochondrial Fo complex, subunit B1 [Source:HGNC Symbol;Acc:HGNC:840] |
|  | ADORA3 | adenosine receptor A3 [Source:RefSeq peptide;Acc:NP_989482] |
|  | RAP1A | RAP1A, member of RAS oncogene family [Source:HGNC Symbol;Acc:HGNC:9855] |
|  | FAM212B | family with sequence similarity 212, member B [Source:HGNC Symbol;Acc:HGNC:28045] |
|  | DDX20 | DEAD (Asp-Glu-Ala-Asp) box polypeptide 20 [Source:HGNC Symbol;Acc:HGNC:2743] |
|  | KCND3 | potassium voltage-gated channel subfamily D member 3 precursor [Source:RefSeq peptide;Acc:NP_989657] |
|  | CTTNBP2NL | CTTNBP2 N-terminal like [Source:HGNC Symbol;Acc:HGNC:25330] |
|  | WNT2B | protein Wnt-2b precursor [Source:RefSeq peptide;Acc:NP_989667] |
|  | ST7L | Suppressor of tumorigenicity 7 protein-like [Source:UniProtKB/Swiss-Prot;Acc:Q90YH8] |
|  | CAPZA1 | F-actin-capping protein subunit alpha-1 [Source:UniProtKB/Swiss-Prot;Acc:P13127] |
|  | MOV10 | putative helicase MOV-10 [Source:RefSeq peptide;Acc:NP_001012861] |
|  | RHOC | Rho-related GTP-binding protein RhoC [Source:UniProtKB/Swiss-Prot;Acc:Q9PSX7] |
|  | PPM1J | protein phosphatase, Mg2+/Mn2+ dependent, 1J [Source:HGNC Symbol;Acc:HGNC:20785] |
|  | SLC16A1 | monocarboxylate transporter 1 [Source:RefSeq peptide;Acc:NP_001006323] |
|  | LRIG2 | leucine-rich repeats and immunoglobulin-like domains 2 [Source:HGNC Symbol;Acc:HGNC:20889] |
|  | MAGI3 | membrane-associated guanylate kinase, WW and PDZ domain-containing protein 3 [Source:RefSeq peptide;Acc:NP_001012715] |
|  | PHTF1 | putative homeodomain transcription factor 1 [Source:HGNC Symbol;Acc:HGNC:8939] |
|  | RSBN1 | round spermatid basic protein 1 [Source:HGNC Symbol;Acc:HGNC:25642] |
|  | PTPN22 | protein tyrosine phosphatase, non-receptor type 22 (lymphoid) [Source:HGNC Symbol;Acc:HGNC:9652] |
|  | BCL2L15 | BCL2-like 15 [Source:HGNC Symbol;Acc:HGNC:33624] |
|  | AP4B1 | AP-4 complex subunit beta-1 [Source:RefSeq peptide;Acc:NP_001026088] |
|  | DCLRE1B | DNA cross-link repair 1B [Source:HGNC Symbol;Acc:HGNC:17641] |
|  | HIPK1 | homeodomain interacting protein kinase 1 [Source:HGNC Symbol;Acc:HGNC:19006] |
|  | OLFML3 | Olfactomedin-like protein 3 [Source:UniProtKB/Swiss-Prot;Acc:Q25C36] |
|  | SYT6 | synaptotagmin VI [Source:HGNC Symbol;Acc:HGNC:18638] |
|  | TRIM33 | tripartite motif containing 33 [Source:HGNC Symbol;Acc:HGNC:16290] |
|  | BCAS2 | pre-mRNA-splicing factor SPF27 [Source:RefSeq peptide;Acc:NP_001244224] |
|  | DENND2C | DENN/MADD domain containing 2C [Source:HGNC Symbol;Acc:HGNC:24748] |
|  | AMPD1 | adenosine monophosphate deaminase 1 [Source:HGNC Symbol;Acc:HGNC:468] |
|  | N-RAS | GTPase NRas [Source:UniProtKB/Swiss-Prot;Acc:Q5F352] |
|  | CSDE1 | cold shock domain-containing protein E1 [Source:RefSeq peptide;Acc:NP_001026089] |
|  | SIKE1 | suppressor of IKBKE 1 [Source:RefSeq peptide;Acc:NP_001006324] |
|  | SYCP1 | synaptonemal complex protein 1 [Source:HGNC Symbol;Acc:HGNC:11487] |
|  | TSHB | Thyrotropin subunit beta [Source:UniProtKB/Swiss-Prot;Acc:O57340] |
|  | TSPAN2 | tetraspanin 2 [Source:HGNC Symbol;Acc:HGNC:20659] |
|  | NGF | Beta-nerve growth factor [Source:UniProtKB/Swiss-Prot;Acc:P05200] |
|  | C1orf74 | chromosome 1 open reading frame 74 [Source:HGNC Symbol;Acc:HGNC:26319] |
|  | gga-mir-205a | gga-mir-205a [Source:miRBase;Acc:MI0001267] |
|  | gga-mir-1669 | gga-mir-1669 [Source:miRBase;Acc:MI0007403] |
| GGA28: 3-5 Mb  pH20  Hb28  Hct28  pCO_2_20  pCO_2_28-20 | NDUFS7 | NADH dehydrogenase (ubiquinone) Fe-S protein 7, 20kDa (NADH-coenzyme Q reductase) [Source:HGNC Symbol;Acc:HGNC:7714] |
|  | GAMT | guanidinoacetate N-methyltransferase [Source:HGNC Symbol;Acc:HGNC:4136] |
|  | DAZAP1 | DAZ-associated protein 1 [Source:RefSeq peptide;Acc:NP_001026599] |
|  | RPS15 | 40S ribosomal protein S15 [Source:UniProtKB/Swiss-Prot;Acc:P62846] |
|  | APC2 | adenomatosis polyposis coli 2 [Source:HGNC Symbol;Acc:HGNC:24036] |
|  | GZMM | granzyme M (lymphocyte met-ase 1) [Source:HGNC Symbol;Acc:HGNC:4712] |
|  | C19orf25 | chromosome 19 open reading frame 25 [Source:HGNC Symbol;Acc:HGNC:26711] |
|  | PCSK4 | proprotein convertase subtilisin/kexin type 4 [Source:HGNC Symbol;Acc:HGNC:8746] |
|  | ADAMTSL5 | ADAMTS-like 5 [Source:HGNC Symbol;Acc:HGNC:27912] |
|  | THOP1 | thimet oligopeptidase 1 [Source:HGNC Symbol;Acc:HGNC:11793] |
|  | SGTA | small glutamine-rich tetratricopeptide repeat-containing protein alpha [Source:RefSeq peptide;Acc:NP_001026550] |
|  | SLC39A3 | solute carrier family 39 (zinc transporter), member 3 [Source:HGNC Symbol;Acc:HGNC:17128] |
|  | MAP1S | microtubule-associated protein 1S [Source:HGNC Symbol;Acc:HGNC:15715] |
|  | GATAD2A | GATA zinc finger domain containing 2A [Source:RefSeq peptide;Acc:NP_001012570] |
|  | MAU2 | MAU2 sister chromatid cohesion factor [Source:HGNC Symbol;Acc:HGNC:29140] |
|  | SUGP1 | splicing factor 4 [Source:RefSeq peptide;Acc:NP_001182480] |
|  | TM6SF2 | transmembrane 6 superfamily member 2 [Source:HGNC Symbol;Acc:HGNC:11861] |
|  | HAPLN4 | hyaluronan and proteoglycan link protein 4 [Source:HGNC Symbol;Acc:HGNC:31357] |
|  | RFXANK | DNA-binding protein RFXANK [Source:RefSeq peptide;Acc:NP_001026560] |
|  | NR2C2AP | nuclear receptor 2C2-associated protein [Source:HGNC Symbol;Acc:HGNC:30763] |
|  | MEF2BNB | protein MEF2BNB isoform 1 [Source:RefSeq peptide;Acc:NP_001243435] |
|  | TMEM161A | transmembrane protein 161A [Source:HGNC Symbol;Acc:HGNC:26020] |
|  | SLC25A42 | solute carrier family 25, member 42 [Source:HGNC Symbol;Acc:HGNC:28380] |
|  | ARMC6 | armadillo repeat containing 6 [Source:HGNC Symbol;Acc:HGNC:25049] |
|  | SUGP2 | SURP and G patch domain containing 2 [Source:HGNC Symbol;Acc:HGNC:18641] |
|  | HOMER3 | homer scaffolding protein 3 [Source:HGNC Symbol;Acc:HGNC:17514] |
|  | DDX49 | probable ATP-dependent RNA helicase DDX49 [Source:RefSeq peptide;Acc:NP_001026109] |
|  | COPE | Coatomer subunit epsilon [Source:UniProtKB/Swiss-Prot;Acc:Q5ZIK9] |
|  | CERS1 | ceramide synthase 1 [Source:RefSeq peptide;Acc:NP_001264694] |
|  | cVg1 | growth differentiation factor 3 [Source:RefSeq peptide;Acc:NP_990542] |
|  | UPF1 | UPF1 regulator of nonsense transcripts homolog (yeast) [Source:HGNC Symbol;Acc:HGNC:9962] |
|  | COMP | cartilage oligomeric matrix protein [Source:HGNC Symbol;Acc:HGNC:2227] |
|  | CRTC1 | CREB regulated transcription coactivator 1 [Source:HGNC Symbol;Acc:HGNC:16062] |
|  | TMEM59L | transmembrane protein 59-like [Source:HGNC Symbol;Acc:HGNC:13237] |
|  | CRLF1 | cytokine receptor-like factor 1 [Source:HGNC Symbol;Acc:HGNC:2364] |
|  | KXD1 | KxDL motif containing 1 [Source:HGNC Symbol;Acc:HGNC:28420] |
|  | FKBP8 | peptidyl-prolyl cis-trans isomerase FKBP8 [Source:RefSeq peptide;Acc:NP_001241668] |
|  | ELL | RNA polymerase II elongation factor ELL [Source:RefSeq peptide;Acc:NP_001012865] |
|  | PGPEP1 | pyroglutamyl-peptidase I [Source:HGNC Symbol;Acc:HGNC:13568] |
|  | LSM4 | LSM4 homolog, U6 small nuclear RNA and mRNA degradation associated [Source:HGNC Symbol;Acc:HGNC:17259] |
|  | PDE4C | phosphodiesterase 4C, cAMP-specific [Source:HGNC Symbol;Acc:HGNC:8782] |
|  | MPV17L2 | MPV17 mitochondrial membrane protein-like 2 [Source:HGNC Symbol;Acc:HGNC:28177] |
|  | GILT | Uncharacterized protein [Source:UniProtKB/TrEMBL;Acc:F1NSR8] |
|  | MAST3 | microtubule associated serine/threonine kinase 3 [Source:HGNC Symbol;Acc:HGNC:19036] |
|  | IL12RB1 | interleukin 12 receptor, beta 1 [Source:HGNC Symbol;Acc:HGNC:5971] |
|  | ARRDC2 | arrestin domain containing 2 [Source:HGNC Symbol;Acc:HGNC:25225] |
|  | C19orf45 | chromosome 19 open reading frame 45 [Source:HGNC Symbol;Acc:HGNC:24745] |
|  | PEX11G | peroxisomal membrane protein 11C [Source:RefSeq peptide;Acc:NP_001006340] |
|  | CTK-1 | Tyrosine-protein kinase receptor [Source:UniProtKB/TrEMBL;Acc:F1NJT2] |
|  | USE1 | unconventional SNARE in the ER 1 homolog [Source:RefSeq peptide;Acc:NP_001026110] |
|  | MYO9B | myosin IXB [Source:HGNC Symbol;Acc:HGNC:7609] |
|  | HAUS8 | HAUS augmin-like complex, subunit 8 [Source:HGNC Symbol;Acc:HGNC:30532] |
|  | CPAMD8 | C3 and PZP-like, alpha-2-macroglobulin domain containing 8 [Source:HGNC Symbol;Acc:HGNC:23228] |
|  | F2RL3 | coagulation factor II (thrombin) receptor-like 3 [Source:HGNC Symbol;Acc:HGNC:3540] |
|  | SIN3B | SIN3 transcription regulator family member B [Source:HGNC Symbol;Acc:HGNC:19354] |
|  | TMEM38A | trimeric intracellular cation channel type A [Source:RefSeq peptide;Acc:NP_001073226] |
|  | SLC35E1 | solute carrier family 35, member E1 [Source:HGNC Symbol;Acc:HGNC:20803] |
|  | CHERP | calcium homeostasis endoplasmic reticulum protein [Source:RefSeq peptide;Acc:NP_001006341] |
|  | C19orf44 | chromosome 19 open reading frame 44 [Source:HGNC Symbol;Acc:HGNC:26141] |
|  | CALR3 | calreticulin 3 [Source:HGNC Symbol;Acc:HGNC:20407] |
|  | EPS15L1 | epidermal growth factor receptor pathway substrate 15-like 1 [Source:HGNC Symbol;Acc:HGNC:24634] |
|  | KLF2 | Kruppel-like factor 2 [Source:UniProtKB/TrEMBL;Acc:E1C442] |
|  | AP1M1 | AP-1 complex subunit mu-1 [Source:RefSeq peptide;Acc:NP_001007887] |
|  | CIB3 | calcium and integrin binding family member 3 [Source:HGNC Symbol;Acc:HGNC:24580] |
|  | RAB8A | Ras-related protein Rab-8A [Source:UniProtKB/Swiss-Prot;Acc:Q5F470] |
|  | TPM4 | tropomyosin 4 [Source:HGNC Symbol;Acc:HGNC:12013] |
|  | PTPRS | receptor-type tyrosine-protein phosphatase delta precursor [Source:RefSeq peptide;Acc:NP_990738] |
|  | KDM4B | lysine (K)-specific demethylase 4B [Source:HGNC Symbol;Acc:HGNC:29136] |
|  | UHRF1 | ubiquitin-like with PHD and ring finger domains 1 [Source:HGNC Symbol;Acc:HGNC:12556] |
|  | DPP9 | dipeptidyl-peptidase 9 [Source:HGNC Symbol;Acc:HGNC:18648] |
|  | C28H19ORF10 | uncharacterized protein LOC420161 precursor [Source:RefSeq peptide;Acc:NP_001006342] |
|  | TNFAIP8L1 | tumor necrosis factor alpha-induced protein 8-like protein 1 [Source:RefSeq peptide;Acc:NP_001006343] |
|  | MED16 | mediator complex subunit 16 [Source:HGNC Symbol;Acc:HGNC:17556] |
|  | R3HDM4 | R3H domain containing 4 [Source:HGNC Symbol;Acc:HGNC:28270] |
|  | DIRAS1 | DIRAS family, GTP-binding RAS-like 1 [Source:HGNC Symbol;Acc:HGNC:19127] |
|  | TICAM1 | TIR domain-containing adapter molecule 1 [Source:RefSeq peptide;Acc:NP_001074975] |
|  | FEM1A | fem-1 homolog a (C. elegans) [Source:HGNC Symbol;Acc:HGNC:16934] |
|  | gga-mir-7-3 | gga-mir-7-3 [Source:miRBase;Acc:MI0001269] |
|  | gga-mir-6666 | gga-mir-6666 [Source:miRBase;Acc:MI0022486] |
|  | gga-mir-1777 | gga-mir-1777 [Source:miRBase;Acc:MI0007520] |
|  | gga-mir-9-1 | gga-mir-9-1 [Source:miRBase;Acc:MI0003694] |
|  | U6 | U6 spliceosomal RNA [Source:RFAM;Acc:RF00026] |
|  | gga-mir-6693 | gga-mir-6693 [Source:miRBase;Acc:MI0022514] |
|  | U6 | U6 spliceosomal RNA [Source:RFAM;Acc:RF00026] |
|  | gga-mir-1621 | gga-mir-1621 [Source:miRBase;Acc:MI0007350] |
|  | gga-mir-1565 | gga-mir-1565 [Source:miRBase;Acc:MI0007290] |
| GGAZ: 5-7 Mb  Glu28  HCO_3_28  TCO_2_28 | KIAA1328 | hinderin [Source:RefSeq peptide;Acc:NP_001026575] |
|  | TPGS2 | tubulin polyglutamylase complex subunit 2 [Source:HGNC Symbol;Acc:HGNC:24561 |
| GGAZ: 69-71 Mb  Glu28  HCO_3_28  TCO_2_28 | CORO2A | coronin, actin binding protein, 2A [Source:HGNC Symbol;Acc:HGNC:2255] |
|  | TBC1D2 | TBC1 domain family, member 2 [Source:HGNC Symbol;Acc:HGNC:18026] |
|  | IKBKAP | inhibitor of kappa light polypeptide gene enhancer in B-cells, kinase complex-associated protein [Source:HGNC Symbol;Acc:HGNC:5959] |
|  | APTX | aprataxin [Source:HGNC Symbol;Acc:HGNC:15984] |
|  | DNAJA1 | dnaJ homolog subfamily A member 1 [Source:RefSeq peptide;Acc:NP_001012963] |
|  | SMU1 | WD40 repeat-containing protein SMU1 [Source:UniProtKB/Swiss-Prot;Acc:Q5ZME8] |
|  | CKI | beta-1,4-galactosyltransferase 1 [Source:RefSeq peptide;Acc:NP_990533] |
|  | SPINK4 | serine peptidase inhibitor, Kazal type 4 [Source:HGNC Symbol;Acc:HGNC:16646] |
|  | HEMGN | hemogen [Source:HGNC Symbol;Acc:HGNC:17509] |
|  | XPA | DNA repair protein complementing XP-A cells homolog [Source:RefSeq peptide;Acc:NP_990184] |
|  | NCBP1 | Nuclear cap-binding protein subunit 1 [Source:UniProtKB/Swiss-Prot;Acc:Q5ZJZ6] |
|  | TSTD2 | thiosulfate sulfurtransferase (rhodanese)-like domain containing 2 [Source:HGNC Symbol;Acc:HGNC:30087] |
|  | TMOD1 | tropomodulin-1 [Source:RefSeq peptide;Acc:NP_990358] |
|  | TDRD7 | tudor domain containing 7 [Source:HGNC Symbol;Acc:HGNC:30831] |
|  | DGKQ | diacylglycerol kinase, theta 110kDa [Source:HGNC Symbol;Acc:HGNC:2856] |
|  | ACO1 | cytoplasmic aconitate hydratase [Source:RefSeq peptide;Acc:NP_001025707] |
|  | FOXE3 | forkhead box E3 [Source:HGNC Symbol;Acc:HGNC:3808] |
|  | SNORA66 | Small nucleolar RNA SNORA66 [Source:RFAM;Acc:RF00155] |
|  | gga-mir-2954 | gga-mir-2954 [Source:miRBase;Acc:MI0013634] |
|  | gga-mir-2131 | gga-mir-2131 [Source:miRBase;Acc:MI0010736] |
|  | gga-mir-1583 | gga-mir-1583 [Source:miRBase;Acc:MI0007309] |

All characterized genes within co-localized QTL for 3 or more traits is listed including QTL location and traits, gene name, and description. Genes were identified using Ensembl Biomart.
